# Mucosal Vaccination with Recombinant *Lactobacillus casei*-Displayed CTA1-Conjugated Consensus Matrix Protein-2 (sM2) Induces Broad Protection against Divergent Influenza Subtypes in BALB/c Mice

**DOI:** 10.1371/journal.pone.0094051

**Published:** 2014-04-08

**Authors:** Mohammed Y. E. Chowdhury, Rui Li, Jae-Hoon Kim, Min-Eun Park, Tae-Hwan Kim, Prabuddha Pathinayake, Prasanna Weeratunga, Man Ki Song, Hwa-Young Son, Seung-Pyo Hong, Moon-Hee Sung, Jong-Soo Lee, Chul-Joong Kim

**Affiliations:** 1 College of Veterinary Medicine (BK21 Plus Program), Chungnam National University, Daejeon, Republic of Korea; 2 Laboratory Science Division, International Vaccine Institute, Seoul, Republic of Korea; 3 BioLeaders Corporation, Daejeon, Republic of Korea; 4 Faculty of Veterinary Medicine, Chittagong Veterinary and Animal Sciences University, Chittagong, Bangladesh; University of Georgia, United States of America

## Abstract

To develop a safe and effective mucosal vaccine against pathogenic influenza viruses, we constructed recombinant *Lactobacillus casei* strains that express conserved matrix protein 2 with (pgsA-CTA1-sM2/*L. casei*) or without (pgsA-sM2/*L. casei*) cholera toxin subunit A1 (CTA1) on the surface. The surface localization of the fusion protein was verified by cellular fractionation analyses, flow cytometry and immunofluorescence microscopy. Oral and nasal inoculations of recombinant *L. casei* into mice resulted in high levels of serum immunoglobulin G (IgG) and mucosal IgA. However, the conjugation of cholera toxin subunit A1 induced more potent mucosal, humoral and cell-mediated immune responses. In a challenge test with 10 MLD_50_ of A/EM/Korea/W149/06(H5N1), A/Puerto Rico/8/34(H1N1), A/Aquatic bird /Korea/W81/2005(H5N2), A/Aquatic bird/Korea/W44/2005(H7N3), and A/Chicken/Korea/116/2004(H9N2) viruses, the recombinant pgsA-CTA1-sM2/*L. casei* provided better protection against lethal challenges than pgsA-sM2/*L. casei*, pgsA/*L. casei* and PBS in mice. These results indicate that mucosal immunization with recombinant *L. casei* expressing CTA1-conjugated sM2 protein on its surface is an effective means of eliciting protective immune responses against diverse influenza subtypes.

## Introduction

Vaccination remains most economical and effective means against respiratory diseases caused by influenza viruses [1]. Based on the circulating viruses in the population, trivalent vaccine strains have been developed and are used for the influenza virus protection [2]. The most acceptable current available strategy is the intramuscular administration of inactivated vaccines produced by egg-based manufacturing systems which while effective, are hampered by limited capacity and flexibility [3]. However, vaccine strains must be frequently adapted to match the circulating viruses throughout the world [4]. In addition, the levels of antibody induced by the inactivated vaccine have been observed to decrease by 75% over an 8-month period [2], [5]. Therefore, alternative strategies for developing broadly cross-protective, safe and effective vaccines against influenza viral infections are of prominent importance.

Matrix protein 2 (M2) is highly conserved among influenza A virus strains, indicating that M2 is an attractive target for developing a universal vaccine [6]. In previous studies, various constructs of the M2 vaccine have been developed and tested, including recombinant *Escherichia coli* (*E. coli*) expressing M2 fusion protein, adenoviral vectors expressing the M2 protein, plasmid DNA encoding M2 [7]–[9] and peptides encoding M2e [11], each of which was able to elicit protective immune responses in mice. However, the drawback of these M2-based vaccines is their low immunogenicity; additionally, most of them would require intramuscular injections. Therefore, many strategies have been applied focusing on increasing the immunogenicity of M2-based vaccines, for example, fusion of M2 with different carrier molecules like human papilloma virus L protein [12], keyhole limpet hemocyanin [10] and flagellin [13]. Furthermore, vaccinations with different adjuvants and routes of administration have been applied to evaluate their protection against divergent strains of influenza viruses. Mice immunized mucosally with an M2 or virus like particles (VLPs) adjuvanted with cholera toxin (CT) demonstrated better protection compared to mice subjected to parenteral immunization [14], [15]. However, due to the adverse effects of CT in humans, investigators have attempted to identify nontoxic subunits with adjuvanticity by removing either subunit A or subunit B [16]. *E. coli* expressing cholera toxin subunit A1 (CTA1) fused with the D-fragment of *Staphylococcus aureus* showed the adjuvant effects without any reactogenicity of the A1 subunit in the mucosal vaccine [6]. Although, chemical or genetic conjugation of M2 may not present M2 in its native tetrameric form, extracellularly accessible antigens expressed on the surfaces of bacteria are better recognized by the immune system than those that are intracellular [17]. Thus, choice of delivery vehicle is also an important concern for potential mucosal vaccines.

Recently, lactic acid bacteria (LAB) presenting influenza virus antigens have been studied [3], [18], [19]. For mucosal immunization, LAB is a more attractive delivery system than other live vaccine vectors, such as *Shigella, Salmonella*, and *Listeria* [20], [21]. It is considered safe and exhibits an adjuvant-like effect on mucosal and systemic immunity [18], [22], [23]. Anchoring of the target protein to the cell surfaces of LAB is primarily intended to use in mucosal vaccines. The transmembrane protein pgsA is one of the poly-γ-glutamate synthetase complexes of *Bacillus subtilis* [17], [24], [25], which is a well-studied anchor protein is able to fuse the target protein to its C terminus and stabilize the complex by anchoring it in the cell membrane. Since sM2 is a highly conserved and promising target for a universal vaccine and CTA1 is strong mucosal adjuvant, in this study, we developed constructs using a consensus sM2 gene reconstituted from the analysis of H1N1, H5N1 and H9N2 influenza viruses (no trans-membrane domain) with or without the fusion of CTA1. To achieve this, we used a novel expression vector that can express a pgsA gene product as an anchoring matrix. Our target antigens, sM2 and CTA1, were displayed on the surface of *Lactobacillus casei*, and the oral or intranasal administration of recombinant *L. casei* induced systemic and mucosal immune responses that have the potential to protect against the lethal challenges of divergent influenza subtypes.

## Materials and Methods

### Animals, Mucosal Immunization and Virus Challenges

A total of 672 female BALB/c mice (5 weeks old) were purchased from Samtako (Seoul, Korea) and housed in ventilated cages. The mice were managed with pelleted feed and tap water *ad libitum*, maintained in a specific-pathogen-free environment and all efforts were made to minimize suffering following approval from the Institutional Animal Care and Use Committee of of Bioleaders Corporation, Daejeon, South Korea, protocol number: BSL-ABLS-13-002. Immunizations of animal were conducted in bio-safety level (BSL)-2 laboratory facilities. Mice were divided into 6 experimental sets, each consisting of 2 subsets: 1 for oral and 1 for intranasal administration which contained 4 groups each. Out of 6, 4 sets had 14 mice per group. One sets had 17 (3 mice for lung histopathology and immunohistochemistry), and the last contained 11 mice per group (3 mice for CTL response).

Concentrations of recombinant *L. casei* were determined by colony forming units (CFU). In each subset, 2 groups received 10^10^ CFU of pgsA-sM2/*L. casei* or pgsA-CTA1-sM2/*L. casei*, and the remaining two groups received the same concentration of pKV-pgsA/*L. casei* or PBS in 100 μl orally via intragastric lavage at days 0 to 3, 7 to 9 and 21 to 23. Similarly, 10^9^ CFU of recombinant cells were administered in 20 μl suspensions into the nostrils of lightly anesthetized mice on days 0 to 3, 7 to 9 and 21. Blood samples were collected from the retro-orbital plexus at days −1, 14 and 28; sera were separated by centrifugation for 5 minutes at 12,000×g and stored at −20°C until analysis. At day 28, 3 mice in each group were randomly sacrificed to collect IgA sample from lungs and intestine and stored at −70°C until analysis. Spleens were collected aseptically at day 28 for the analysis of the CTL response randomly from 3 mice of one set. The rest of the mice from the same set were maintained for 6 months from the date of the last boosting to measure the long-lasting immune responses and protection efficacy.

The avian influenza viruses A/EM/Korea/W149/06(H5N1), A/Puerto Rico/8/34(H1N1), A/Aquatic bird/Korea/W81/2005 (H5N2), A/Aquatic bird/Korea/W44/2005(H7N3), and A/Chicken/Korea/116/2004(H9N2) used in this study were kindly provided by Dr. Young-Ki Choi (College of Medicine and Medical Research Institute, Chungbuk National University, Cheongju, Republic of Korea). All viruses were propagated in the allantoic fluid of 10-day-old chicken embryos, and 50% mouse lethal doses (MLD_50_) were determined in 8-week-old naive BALB/c mice. Ether narcosis-anesthetized mice were intranasally infected with 10 times the MLD_50_ of challenge viruses in 20 μl of PBS. Six mice in each group were sacrificed on 3 and 5 dpi to check virus titer in lungs and other 5 mice remained in each group have been used for survival. Mice were monitored every alternate day at fixed time point for measuring the weight loss and survival. Mice were euthanized if moribund, i.e. weight loss, ruffled fur, shivering, tachypnea, respiratory distress, hypothermia and poorly responsive to external stimuli, remaining were considered as survival number. After final monitoring, all the survived mice were humanely euthanized using CO_2_ inhalation for 5 minutes.

At 180 days after the final vaccination, mice from one set were challenged with H5N2 for measuring the long lasting immune responses. All challenge tests were conducted inside an approved BSL-3+ facility under appropriate conditions.

### Bacterial Strains and Cloning for the Construction of Recombinant Plasmid PgsA-sM2/*L. casei* and PgsA-CTA1-sM2/*L. casei*

In this study, *E. coli* JM83 was used for cloning and *L. casei* L525 was used for surface expression of the target protein. These bacteria were grown in LB and MRS media, respectively. The plasmid pKV-Pald-PgsA, harboring the pgsA genes of *Bacillus subtilis*, was used to construct the surface display plasmid, which was a kind gift from the Bioleaders Corporation (Daejeon, South Korea). A gene encoding the consensus sequence of M2 spanning the residues of the extracellular and cytoplasmic domains without the transmembrane domain of influenza virus was generated. The consensus sequences were created based on the most common amino acids in each position of the alignment of H1N1, H5N1 and H9N2; then, they were synthesized and used as templates for the construction of the plasmids pgsA-sM2/*L. casei* and pgsA-CTA1-sM2/*L. casei* by cloning, as described previously [26], [27]. The sM2 gene was modified by adding a *Kpn* I site at the 5′ terminal and *Sal* I at the 3′ terminal for cloning. The polymerase chain reaction (PCR) was performed to amplify the gene using the primer pair 5′-GGGGTACCTCATTATTAACA-3′, and 5′-ACGTCGACTCATTATTCAAGTTCAATAATG AC-3′. Similarly, a *Bam*H I site at the 5′ terminal and a *Kpn* I site at the 3′ terminal end were added to the CTA1 gene using primers 5′-CGGGATCCAATGATGATAAGTTATAT-3′ and 5′-GGGT ACCCGATGATCTTGGAGC ATT-3′. The modified genes were ligated into the T Easy Vector (Invitrogen, Seoul, Korea). Genes were then digested with *Kpn* I-*Sal* I for sM2 and *Bam*H I-*Kpn* I for CTA1. The digested sM2 was ligated to the plasmid vector pKV-pgsA for the construction of pKV-pgsA-sM2. Similarly, CTA1 was ligated for the construction of pKV-pgsA-CTA1-sM2. The ligated products were transformed into *E. coli* JM83 competent cells, as previously described, using an electroporation method [17]. The profiles of the recombinant plasmids were confirmed by restriction endonuclease digestion and DNA sequencing (Solgent, Seoul, Korea). After confirmation, the plasmids were transformed into *L. casei* L525 by electroporation and named pgsA-sM2/*L. casei* and pgsA-CTA1-sM2/*L. casei.*

### Cell Fractionation and Immunoblotting

The recombinant *L. casei* containing pgsA, pgsA-sM2 and pgsA-CTA1-sM2 genes were grown at 30°C for 48 hours. Cells were harvested by centrifugation at 6,000×g for 10 minutes at 4°C, followed by washing two times with sterile phosphate-buffered saline (PBS). Bacterial lyses were performed by sonication and centrifuged at 12,000×g for 20 minutes at 4°C. Cell wall and cytoplasmic fractions were separated by centrifugation at 25,000×g at 4°C for 2 hours. Pellets (cell wall) were resuspended in 100 μl of 1% sarcosol containing 1 mM phenylmethylsulfonyl fluoride (PMSF, Sigma-Aldrich, St. Louis, USA) as a protease inhibitor. Fractions were analyzed by western blotting, as described previously. For the immune detection of fusion proteins, the membranes were probed with rabbit anti-cholera toxin (1∶2000, Abcam, UK), rabbit anti-pgsA (1∶1000) and rabbit anti-M2 (1∶1000) antibodies. The rabbit anti-pgsA and rabbit anti-M2 antibodies used in this experiment were generated by the i.m. inoculation of KLH-conjugated pgsA or M2 peptide in rabbit, respectively, two times at 2 weeks-interval. The membranes were reacted with a 1∶10,000 dilution of anti-rabbit immunoglobulin G conjugated with horseradish peroxidase (IgG HRP). Finally, the target proteins were detected using the WEST-ZOL plus Western Blot Detection System (iNtRON Biotechnology, Gyeonggi-do, Korea) and visualized by enhanced chemiluminescence (ECL) [17], [26], [28].

### Flow Cytometry and Immunofluorescence Microscopy

To investigate the expression of sM2 or CTA1-sM2 on the surface of *L. casei*, recombinant *L. casei* were grown in 30°C for 48 hours in the MRS broth. Bacteria were harvested by centrifugation at 5,000×g for 10 minutes at 4°C, washed three times with sterile phosphate-buffered saline containing 0.01% Tween-20 (PBST) and probed with polyclonal rabbit anti-M2 or rabbit anti-CT antibody overnight. Following another washing, the cells were treated with fluorescein isothiocyanate (FITC)-conjugated anti-rabbit IgG antibodies (Burlingame, CA, USA) for 2 hours. Finally, 10,000 cells were analyzed by flow cytometry (Becton Dickinson, Oxnard, CA, USA). For the immunofluorescence, cells were prepared under the same condition described for the flow cytometry. The pgsA/*L. casei* was used as a negative control and Immunofluoresence analysis was examined using a Carl Zeiss Axioskop 2 fluorescence microscope.

### ELISA

Antibody titers were measured by enzyme-linked immunosorbent assay (ELISA) using serum or mucosal samples from vaccinated mice. First, 96-well immunosorbent plates (Nunc) were incubated with 300 ng/well purified sM2 or CTA1 proteins at 4°C overnight. The recombinant sM2 and CTA1 proteins used in this study were purified from *E. coli*. Next, the wells were blocked with 10% skim milk for 2 hours in RT, washed five times with PBST, treated with diluted serum samples (1∶200) in triplicate for detecting IgG and undiluted tissue homogenized supernatant for detecting local IgA and incubated for 2 hours at 37°C. After washing three times, goat anti-mouse IgG HRP (1∶1000, sigma) or anti-mouse IgA was added to each well and incubated for an additional 2 hours at 37°C. Following another round of washing, the plates were reacted with the substrate solution containing tetramethylbenzidine and H_2_O_2_ and allowed to precede the reaction for 10 minutes. After adding the stop solution 2N-H_2_SO_4_, the optical density (OD) was measured at 450_nm_ using an ELISA autoreader (Molecular devices).

### ELISPOT Assay

The development and counting of cytokines were performed by ELISPOTs, as described previously [31], [32]. Briefly, the day before the isolation of splenocytes, ELISPOT 96-well plates were coated with monoclonal anti-mouse IFN-γ and IL-4 capture antibodies (5 μg/ml) in PBS and incubated at 4°C overnight. The plates were washed with PBS, and 200 μl/well of blocking solution containing complete RPMI 1640 medium and 10% fetal bovine serum, was added (Invitrogen, Carlsbad, CA, USA) and incubated for 2 hours in RT. Spleens from the vaccinated mice were isolated aseptically and added at 5×10^4^ cells/well in media containing sM2 protein, M2 peptide (SLLTEVETPTRNGWECKCSD) (1 μg/well), only medium (negative control), or 5 μg/ml phytohemagglutinin (positive control, Invitrogen, Carlsbad, CA, USA). After adding cells and stimulators, the plates were incubated for 24 hours at 37°C with 5% CO_2_. The plates were sequentially treated with biotinylated anti-mouse IFN-γ and IL-4 antibodies, streptavidin-horseradish peroxidase, and substrate solution. Finally, the spots were counted using an ImmunoScan Entry analyzer (Cellular Technology, Shaker Heights, USA).

### Lungs Virus Titer

The lungs were collected aseptically, and virus titers were determined by 50% tissue culture infectious dose (TCID_50_), as described previously [33]. Briefly, lung tissues were homogenized in 500 μl of PBS containing antibiotics (penicillin, and streptomycin) and antimycotics (Fungizone) compounds (Gibco, Grand Island, NY, USA). Mechanically homogenized lung samples were centrifuged (15 minutes, 12,000×g and 4°C) to remove the cellular debris before their storage at −80°C. MDCK cells were inoculated with a 10-fold serially diluted sample and incubated at 37°C in a humid atmosphere of 5% CO_2_ for an hour. After absorption, the media was removed, and overlay medium containing L-1-tosylamido-2-phenylethyl chloromethyl ketone (TPCK) trypsin (Thermo Fisher Scientific, Rockford, USA) was added to the infected cells and incubated for 72 hours. Viral cytopathic effects were observed daily, and the titers were determined by the HA test. The viral titer of each sample was expressed as 50% tissue infected doses using the Reed-Muench method [34].

### Histopathology and Immunohistochemistry

For histopathology, lung tissues were collected at 5 dpi from ether narcosis-anesthetized mice. Tissues were immediately fixed in 10% formalin containing neutral buffer, embedded in paraffin wax, sectioned at 4–6 μm thickness using a microtome machine, mounted onto slides, and stained with eosin stain. Histopathological changes were examined by light microscopy, as previously described [29], [30], [35]. Furthermore, slides were stained using an immunoperoxidase method with an antibody (rabbit anti-M2, 1∶500) directed against the matrix protein-2 of influenza A virus. A Goat-anti-rabbit IgG HRP (1∶2000, Sigma-Aldrich, St. Louis, USA) was used as the secondary antibody for the detection of virus infected cells in respective tissues [57].

### Statistics

Data are presented as the means ± standard deviations (S.D.) and are representative of at least three independent experiments. Differences between groups were analyzed by analysis of variance (ANOVA), and means were compared by Student's *t*-test. *P*-values less than 0.05 were regarded as significant. Results for percent initial body weight were also compared by using Student's *t* test. Comparison of survival was done by log-rank test using GraphPad Prism 6 version.

## Results

### Construction of Plasmids Containing sM2 with or without CTA1 and Expression on *L. casei*

**Figure 1:**
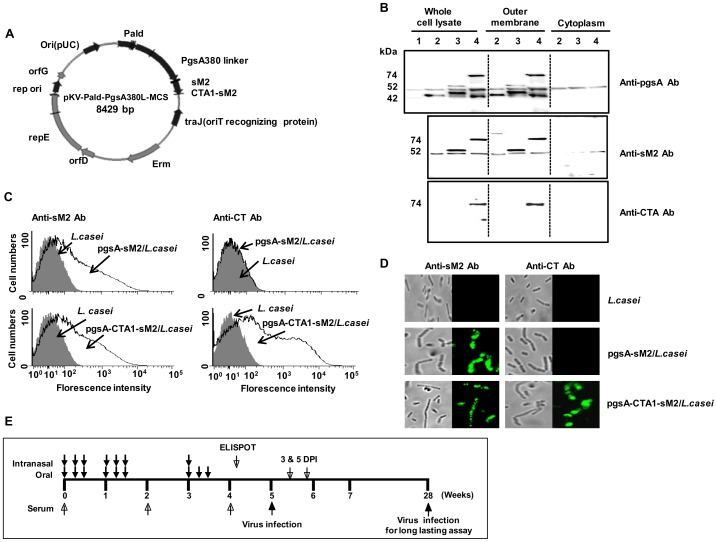
Construction and surface expression of sM2 and CTA1-conjugated sM2 protein on *Lactobacillus casei*. (A) A schematic diagram of the pgsA-sM2 and pgsA-CTA1-sM2 plasmids. (B) Western blot analysis of recombinant pgsA-sM2/*L. casei* and pgsA-CTA1-sM2/*L. casei* expression using anti-pgsA (top), anti-M2 (middle) and anti-CT (bottom) polyclonal antibodies. Lanes 1 and 2 show *L. casei* and *L. casei* with the parental vector (pgsA/*L. casei*), respectively. Lanes 3 and 4 show pgsA-sM2 and pgsA-CTA1-sM2, respectively. Protein bands of 42, 52 and 74 kDa, corresponding to pgsA, pgsA-sM2 and pgsA-CTA1-sM2, respectively, were detected in lanes 2, 3 and 4 of the whole-cell lysates (left) and outer membrane fractions (middle) but not in the cytoplasm (right). (C) Fluorescence-activated cell sorting (FACS) analysis of *L. casei* (filled) and recombinant *L. casei* (open) cells. The cells were reacted with either rabbit anti-sM2 (left) or anti-CT (right) polyclonal antibodies, followed by reaction with a fluorescence isothiocyanate-conjugated (FITC) anti-rabbit IgG antibody. (D) Representative immunofluorescence images of *L. casei* (control) and recombinant *L. casei* cells expressing sM2 and CTA1-sM2. The cells were treated with anti-M2 or anti-CT and then FITC-conjugated anti-rabbit IgG antibodies. Bright-field images are shown on the right. (E) Mouse vaccination and virus challenge experimental protocols. The mice were grouped as mentioned in materials and methods and received oral or nasal administrations, according to the schedule. Arrows indicated the immunization routes and periods of pgsA/*L. casei*, pgsA-sM2/*L. casei* or pgsA-CTA1-sM2/*L. casei* cells. Sera were collected at days 0, 14 and 28; samples from the lungs and intestines were collected at day 28 after immunization. A week after the final immunization, spleens were excised from 3 mice in each group, with one set for CTL analysis. Two or 24 weeks after the last immunization, all mice were challenged with a lethal dose of influenza subtypes through intranasal route and monitored for 13 days. On days 3 and 5 post infection, the lungs were excised from 3 mice in each group to determine the virus titer. On 5 dpi, the mice from one set were sacrificed for lung histopathology and immunohistochemistry.

The pgsA-expressing vector was used to construct plasmids containing the highly conserved consensus sM2 gene, with (pgsA-CTA1-sM2) or without (pgsA-sM2) the cholera toxin subunit A1 (CTA1, Fig. 1A). Plasmids were transformed into *L. casei* cells. The expression levels of pgsA-sM2 and pgsA-CTA1-sM2 were monitored by immunoblotting using anti-pgsA, anti-M2 or anti-CT polyclonal antibodies (data not shown).

To determine the cellular localization of the sM2 and CTA1 proteins expressed on the surface of *L. casei* via the cell wall anchor protein pgsA, membrane and cytoplasmic fractions were subjected to western blot analysis. As expected, both pgsA-sM2 and pgsA-CTA1-sM2 fusion proteins were detected by anti-pgsA, anti-M2 or anti-CT polyclonal antibodies in the membrane, not in cytoplasmic fractions (Fig. 1B, lane 2, 3 and 4). Immunoreactions were performed with anti-pgsA, and bands representing the size of the fused proteins pgsA-sM2 and pgsA-CTA1-sM2 were detected, while during the reactions with anti-M2 or anti-CT antibodies, no other bands were detected (Fig. 1B, lane 3 and 4). This finding may have resulted from the degradation that occurs during the membrane fractionation procedure.

Fluorescence-activated cell sorting (FACS) and immunofluorescence labeling of the cells were used to verify the localization of the fusion pgsA-sM2 and pgsA-CTA1-sM2 protein on the surface of *L. casei*. Flow cytometric analysis using rabbit anti-M2 and anti-CT antibodies revealed increase level of fluorescence intensity of pgsA-sM2/*L. casei* or pgsA-CTA1-sM2/*L. casei* cells, compared to that of control *L. casei* cells (Fig. 1C). Immunofluorescence microscopy also showed recombinant bacteria harboring pgsA-sM2 or pgsA-CTA1-sM2 that immunostained positive for sM2 and CTA1, but this was not found in control cells. These results demonstrated that recombinant *L. casei* could efficiently display the sM2 and CTA1-sM2 fusion proteins on the surface, using pgsA as a membrane anchor protein.

### Immune Responses Induced by Mucosal Immunization with *L. casei* Surface Displayed sM2 and CTA1-sM2

**Figure 2:**
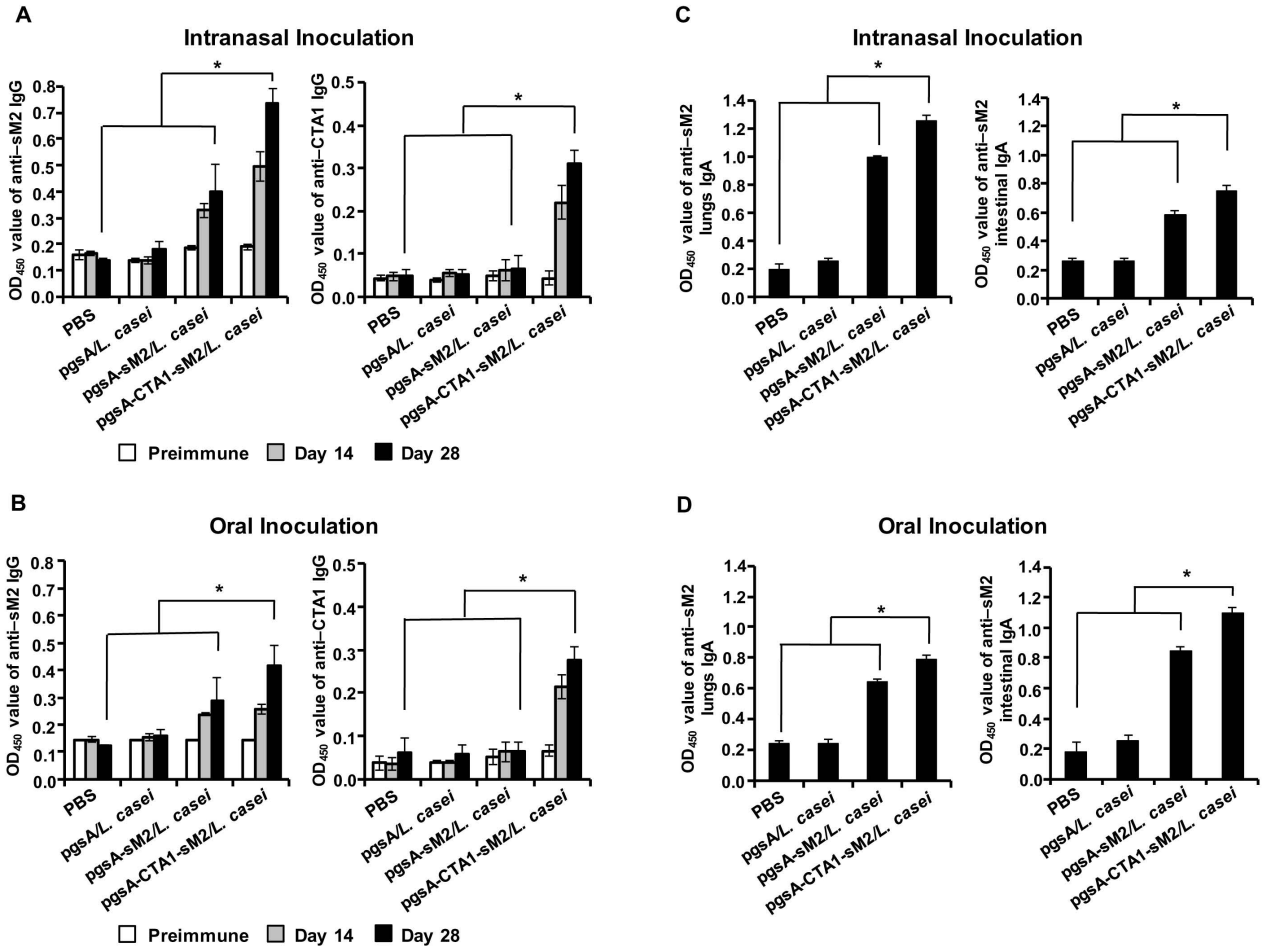
Evaluation of the immune responses after the administration of recombinant *L. casei* in Balb/c mice. Mice were immunized orally or intranasally with PBS, pgsA/*L. casei*, pgsA-sM2/*L. casei* or pgsA-CTA1-sM2/*L. casei* at 0 to 3, 7 to 9 and 21 to 23 days orally or 0 to 3, 7 to 9 and 21 days intranasally. Sera were collected on days 0, 14, and 28. Mouse lungs and intestines were collected at day 28. (A and B) The absorbance of the IgG antibodies specific to the sM2 (left) and CTA1 (right) proteins in the intranasal (top) and oral (bottom) groups. (C and D) The absorbance of the IgA antibody specific to the sM2 protein in the lungs (left) and intestines (right) in the intranasal (top) and oral (bottom) groups. Serum IgG, lung IgA and intestinal IgA were investigated by ELISA and measured at an absorbance of 450_nm_. Bars denote mean ± S.D. The asterisk indicates a significant difference between pgsA-CTA1-sM2/*L. casei* and other groups (*P<0.05).

Preliminary experiment was conducted to determine the doses and schedule of pgsA-CTA1-sM2/*L. casei* vaccine candidate on influenza virus protection (data not shown). To characterize the immunogenicity of the *L. casei* surface-displayed sM2 and CTA1-conjugated sM2, BALB/c mice were immunized nasally (10^9^ cells/20 μl dose) or orally (10^10^ cells/100 μl dose) with recombinant live pgsA-sM2/*L. casei* and pgsA-CTA1-sM2/*L. casei* bacteria. As a negative control, mice were immunized with *L. casei* harboring the parental plasmid pKV-pgsA (pgsA/*L. casei*) and PBS. Serum samples were collected at 0, 14 and 28 days and analyzed by ELISA, using sM2 and CTA1 proteins (purified from *E. coli*) as a coating antigen. After the first series of immunization, comparatively low levels of serum IgG were detected both in the i.n. and orally immunized group. However, high antibody levels were detected shortly after the second series of immunization, and the CTA1-conjugated sM2 group induced serum IgG at significant level, compared to sM2-only group and negative controls (Fig. 2A and B). Although the conjugation of CTA1 with sM2 was expected to have an adjuvant function only, a significant level of anti-CTA1 antibodies was detected in both the nasal and oral vaccinations (Fig. 2A and B right panel). In comparison with the oral group, the nasally immunized group showed higher levels of serum IgG specific to both sM2 and CTA1.

To assess the mucosal immune responses, the local IgA levels were determined by ELISA. Lung and intestinal tissues were collected at day 28 of immunization and examined using sM2 protein as a coating antigen. In both routes of vaccination, pgsA-CTA1-sM2/*L. casei* induced significantly increased levels of sM2-specific mucosal IgA compared to the pgsA-sM2/*L. casei* and control groups. However, as expected, higher levels of antibody titers were detected at the site of inoculation than at the remote site. A similar pattern of antibody responses was observed for both routes of immunization, in which the pgsA-CTA1-sM2/*L. casei* groups dominated (Fig. 2C and D). These data demonstrated that cholera toxin subunit A1-conjugated sM2 resulted in significant enhancements to the sM2-specific IgG and mucosal IgA levels compared with sM2 alone or with controls immunized with pgsA/*L. casei* or PBS.

### Mucosal Immunization with *L. casei* Surface-displayed sM2 and CTA1-sM2 Stimulated M2-specific Cellular Immune Response

**Figure 3:**
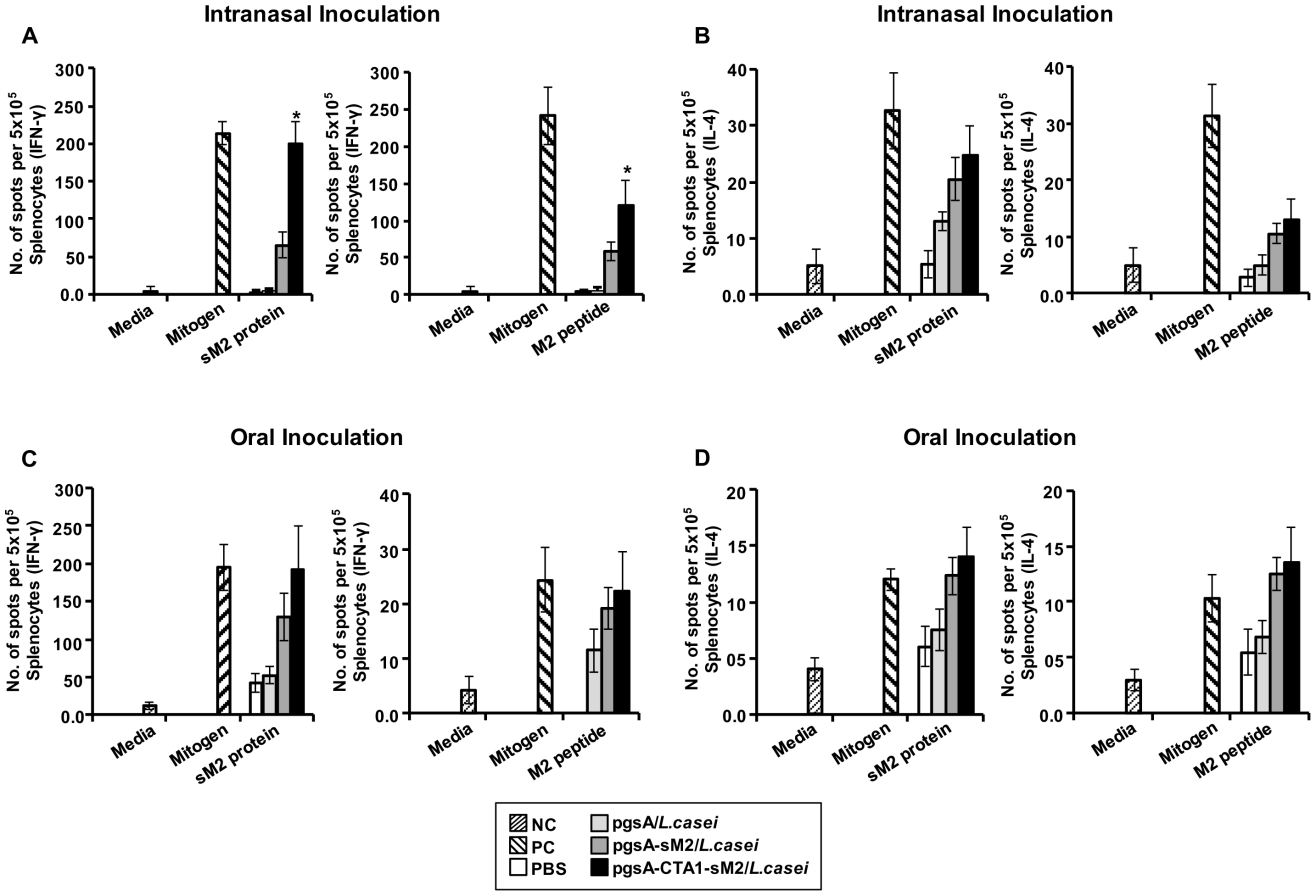
Determination of cytokine-producing splenocytes. Cytokines (IFN-γ and IL-4) were enumerated by ELISPOT at 7 days after the final immunization from the splenocytes. (A and C) Number of spots specific to IFN-γ per 5×10^5^ splenocytes were stimulated with sM2 proteins (left) and M2 peptides (right) of the intranasally (top) and orally (bottom) immunized groups. (B and D) Number of spots specific to IL-4 per 5×10^5^ splenocytes stimulated with sM2 protein (left) and M2 peptide (right) of the intranasally (top) and orally (bottom) immunized groups. NC denotes negative controls (non-immunized), and PC denotes positive controls, the cells stimulated with mitogen. Bars denote mean ± S.D. The asterisk indicates a significant difference between pgsA-CTA1-sM2/*L. casei* and other groups (*P<0.05).

To determine whether mucosal vaccination with *L. casei* surface-displayed sM2 and CTA1-conjugated sM2 could induce cellular immunity, IFN-γ and IL-4 ELISPOT were performed. Splenocytes from vaccinated mice were stimulated with 10 μg/ml of recombinant sM2 protein or M2 peptide, and the cytokine ELISPOTs were developed. The spots were counted to measure the differences in the CTL responses between the groups. Cells from the mice immunized i.n. with pgsA-CTA1-sM2/*L. casei* showed significant levels of IFN-γ in response to stimulation with sM2 protein and M2 peptide (Fig. 3A). Similarly, we observed that i.n. administered groups both for pgsA-sM2/*L. casei* and pgsA-CTA1-sM2/*L. casei* showed detectable levels of IL-4 secreting splenocytes following stimulation with either sM2 protein or M2 peptide (Fig. 3B). IFN-γ and IL-4 secreting cells were also observed in mice immunized orally with pgsA-sM2/*L. casei* and pgsA-CTA1-sM2/*L. casei* (Fig. 3C) although their levels were lower than i.n. group and were not significant. Control group immunized with pgsA/*L. casei* showed background spot level for both in intranasal and oral groups. These findings indicate that highly conserved sM2 can induce M2-specific IFN-γ and IL-4 secreting T cell responses, while mucosal delivery through *L. casei* and CTA1 conjugation with sM2 enhanced the cell mediated immunity, which may contribute to broadening the protective immunity.

### Protection against Challenges with Divergent Influenza Virus Subtypes Containing Heterologous sM2 Sequence

**Figure 4:**
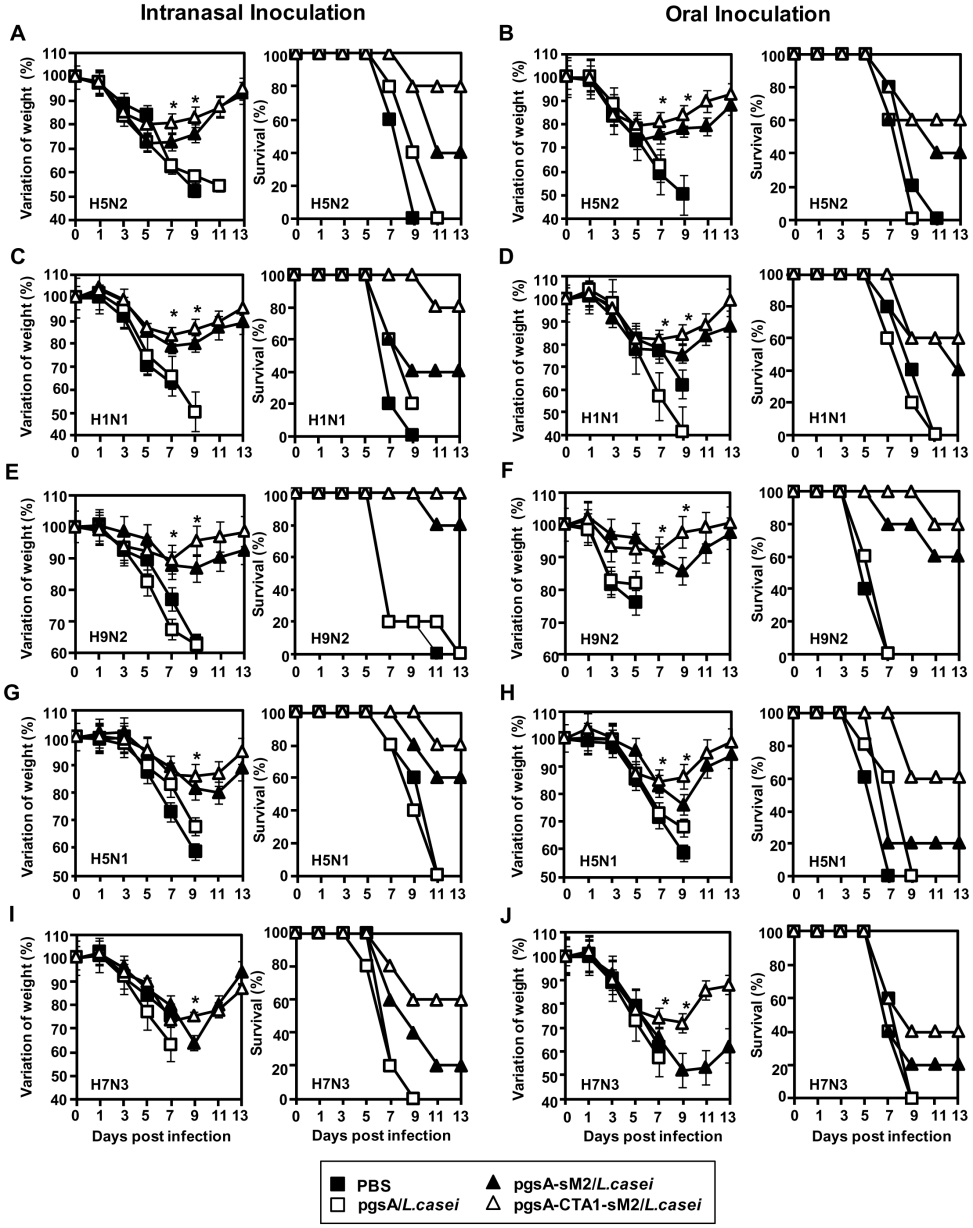
Protection efficacy of recombinant LAB against infections with divergent influenza virus subtypes. Five-week-old female BALB/c mice (5 per group) were vaccinated with PBS, pgsA/*L. casei*, pgsA-sM2/*L. casei* or pgsA-CTA1-sM2/*L. casei* at 0 to 3, 7 to 9 and 21 days i.n. or 0 to 3, 7 to 9 and 21 to 23 days orally. Vaccinated mice were infected i.n. with 10 LD_50_ of mouse-adapted divergent influenza subtypes. (A-J left panel) The percent change of initial body weight and, (A-J right panel) survival post challenge are shown. (A-J left panel) Error bars depict the standard error of the mean. *, Groups differ for weight loss, P<0.05 (Student's *t* test). In survival, mice vaccinated (particularly i.n. group) with pgsA-CTA1-sM2/*L. casei* was significantly better protected than PBS, pgsA/*L. casei*, pgsA-sM2/*L. casei* mice after lethal challenge with (A and B, right panel) A/Aquatic bird/Korea/W81/2005(H5N2) (log-rank test, *P* = 0.00063 and *P* = 0.045 respectively), (C and D, right panel) A/Puerto Rico/8/34(H1N1) (log-rank test, *P* = 0.01 and *P* = 0.02 respectively), (E and F, right panel) A/Chicken/Korea/116/2004(H9N2) (log-rank test, *P* = 0.0004 and *P* = 0.004 respectively), (G and H, right panel) A/EM/Korea/W149/06(H5N1) (log-rank test, *P* = 0.001 and *P* = 0.06 respectively) and (I and J, right panel) A/Aquatic bird/Korea/W44/2005(H7N3) (log-rank test, *P* = 0.02 and *P* = 0.56 respectively).

M2 is known as a potential target for the development of broad spectrum influenza vaccine with minimum variability [36], [37]. To confirm the variability of sM2 sequences of the challenged viruses used in this study, we compared the sM2 of influenza subtypes available from U.S. National Center for Biotechnology Information (NCBI) with our consensus sM2 sequence particularly the whole conserved ecto and some portion of cytoplasmic domain (CD) although entire CD was included in vaccine construct (Table 1). We found that, viruses used in this study contain 0–8 mismatched amino acids among the amino acids of sM2 compared in this study. To evaluate the efficacy of the sM2 vaccine, week after the final immunization, mice were challenged i.n. with the 10 MLD_50_ of A/Aquatic bird/Korea/W81/2005 (H5N2) influenza virus subtypes that was homologous to the consensus sM2 sequence. Mice immunized orally with pgsA-sM2/*L. casei* and pgsA-CTA1-sM2/*L. casei* showed 40 and 60% protection respectively. Similarly, i.n. immunization groups conferred 40 and 80%, against the lethal infection with highly virulent H5N2 virus. In contrast, none of the unimmunized mice survived after lethal infection (Fig. 4A and B, right panel). Morbidity was increased in the mice immunized via oral route, whereas mice that received i.n. immunization with pgsA-CTA1-sM2/*L. casei* lost <20% of their initial body weight and started recovering by 9 day post infection (dpi) and had completely recovered by day 13 (Fig. 4A and B, left panel).

**Table 1:** Comparison of the sM2 sequence between the vaccine and challenge strains.

Virus strain	Subtype	Amino acid sequence	Access no.
Consensus		MSLLTEVETPTRNGWECKCSDSSDPDRLFFKCIYRRLKYGLKRGPSTEGV	
A/EM/Korea/W149/06	H5N1	MSLLTEVETPTRN**E**WECRCSDSS**D**PDRLFFKCIYRRLKYGLKRGPSTEGV	ABW73743.1
A/Aquaticbird/Korea/W81/2005	H5N2	MSLLTEVETPTRNGWECKCSDSS**D**PDRLFFKCIYRRLKYGLKRGPSTEGV	EU819138.1
A/Puerto Rico/8/34	H1N1	MSLLTEVETPIRN**E**W**G**C**R**C**NG**SS**D**PDRLFFKCIYRR**F**KYGLK**G**GPSTEGV	NC_002016.1
A/Aquaticbird/Korea/W44/2005	H7N3	MSLLTEVETPTRNGWEC**R**CSDSS**D**PDRLFFKCIYRRLKYGLKRGPSTEGV	JN244137.1
A/Chicken/Korea/116/2004	H9N2	MSLLTEVETPTRNGWECKCSDSS**D**P**L**RLFFKCIYRRLKYGLKRGPSTEG**M**	EU819104.1

Amino acids that differ from consensus sM2 sequences are in bold and underlined.

We next evaluated the protection efficiency of sM2 vaccine candidate against A/Puerto Rico/8/34(H1N1), which contains 8 mismatched amino acids relative to the sM2 consensus sequence. Sets of vaccinated mice were challenged with 10 MLD_50_ of the H1N1 virus. As shown in figure 4C and D, mice immunized by the i.n route exhibited a higher level of protection than the orally immunized groups, and mice immunized with pgsA-CTA1-sM2/*L. casei* showed a significantly higher level of protection compared to mice immunized with pgsA-sM2/*L. casei* (Fig. 4C and D, right panel). Unimmunized mice lost up to 40% of their body weight and died by 9 dpi. Mice immunized with pgsA-CTA1-sM2/*L. casei* lost approximately 10% of their body weight, whereas mice immunized with pgsA-sM2/*L. casei* lost >20% of their initial body weight by 9 dpi and recovered more slowly than mice immunized with pgsA-CTA1-sM2/*L. casei* (Fig. 4C and D, left panel).

Another set of vaccinated mice were infected with A/Chicken/Korea/116/2004(H9N2) to check the range of protection ability of sM2 vaccine induced immune responses. The sM2 sequence of H9N2 contains 2 mismatched relative to the sM2 consensus sequence. The mice immunized with pgsA-CTA1-sM2/*L. casei* showed negligible body weight losses and gradual recovery compared to those of mice immunized with pgsA-sM2/*L. casei* and the unimmunized mice for both the i.n and oral routes (Fig. 4E and F left panel). None of the unimmunized mice survived, whereas 100% and 80% of the mice immunized with pgsA-CTA1-sM2/*L. casei* via the i.n. and oral routes survived, respectively. The survival rates of mice immunized with pgsA-sM2/*L. casei* were 80% and 60% for the i.n. and oral routes, respectively (Fig. 4E and F, right panel).

The breadth of protection of the sM2 vaccine against divergent influenza subtypes was also evaluated. Set of immunized mice were challenged with high pathogenic avian influenza (HPAI) A/EM/Korea/W149/06(H5N1), which contains 2 amino acid mismatches relative to the sM2 consensus sequence. Mice immunized via the i.n. and oral routes with pgsA-CTA1-sM2/*L. casei* showed higher protection efficacies, 80% and 60%, respectively, compared with mice immunized with pgsA-sM2/*L. casei*, for which the rates were 60% and 20%, respectively (Fig. 4G and H, right panel). Regarding morbidity, mice immunized with pgsA-CTA1-sM2/*L. casei* showed lower morbidity than mice immunized with pgsA-sM2/*L. casei* (Fig. 4G and H, left panel). One more set of vaccinated mice were challenged with the A/Aquatic bird/Korea/W44/2005 (H7N3) virus, which contains 1 mismatch relative to the consensus sM2 sequence, and the body weight and survival were observed for 13 dpi. As shown in figure 4I and J, unimmunized mice lost as much as 30% of their body weight than mice immunized with pgsA-sM2/*L. casei* and pgsA-CTA1-sM2/*L. casei* (Fig. 4I and J, left panel). Mice immunized with pgsA-CTA1-sM2/*L. casei* through the i.n route showed significantly higher level of protection against the H7N3 influenza virus than the other groups (Fig. 4I and J, right panel). Taken together, the results indicate that i.n. immunization with pgsA-CTA1-sM2/*L. casei* induced immune responses that conferred significant levels of protection against divergent subtypes of influenza viruses containing mismatched amino acids ranging from 0 to 8 of the consensus sM2, regardless of whether it was complete or partial.

### Lung Virus Titers and Histopathology

**Figure 5:**
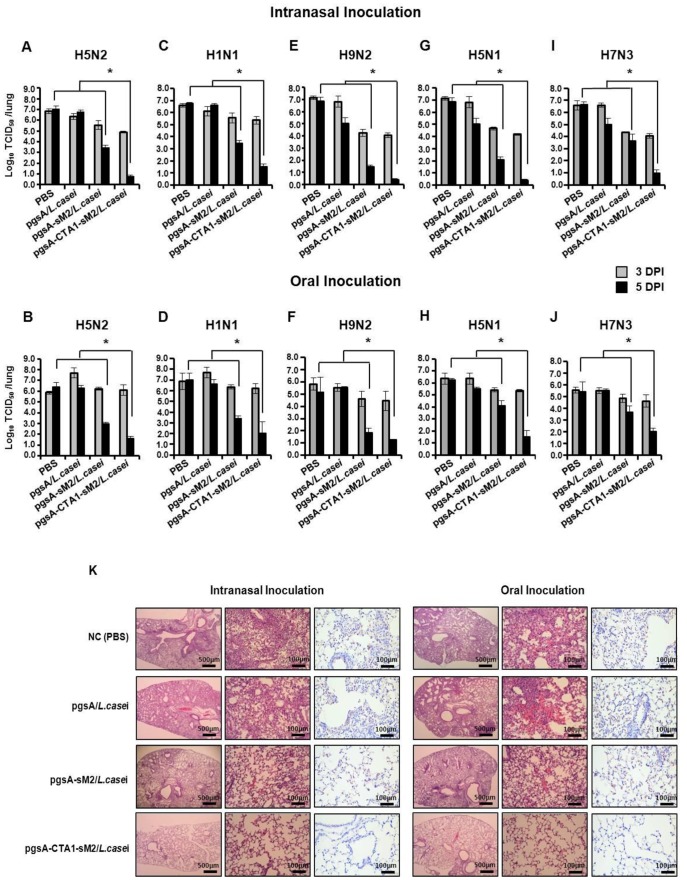
Lung virus titers, histopathology and immunohistochemistry. Virus titers in the lung tissues were determined by TCID_50_ in the MDCK cell line at 3 and 5 dpi after challenge with (A and B) A/Aquatic bird Korea/W81/2005(H5N2), (C and D) A/Puerto Rico/8/34(H1N1), (E and F) A/Chicken/Korea/116/2004 (H9N2), (G and H) A/EM/Korea/W149/06(H5N1) and (I and J) A/Aquatic bird/Korea/W44/2005(H7N3) influenza subtypes. (K) The lungs of the mice vaccinated with pgsA-CTA1-sM2/*L. casei* showed clear alveoli without inflammatory cell infiltration, in contrast to the lungs of mice vaccinated with pgsA-sM2/*L. casei* or control mice, both of which revealed features of severe pneumonitis (middle and left panel). Reduced number of viral antigen were detected in lungs of the mice vaccinated with pgsA-CTA1-sM2/*L. casei*, in contrast to the lungs of mice vaccinated with pgsA-sM2/*L. casei* or control revealed features of severe pneumonitis with increase virus antigen (right panel). Micrographs are representative for each treatment group at a magnification of 200X. Virus antigen in epithelial cells appears as brown coloration of the nucleus and cytoplasm. In lung titers, bars denote mean ± S.D. The asterisk indicates a significant difference between pgsA-CTA1-sM2/*L. casei* and other groups (*P<0.05).

Virus titers in the lungs of challenged mice were measured to estimate replication at 3 and 5 dpi. Mice were immunized via the i.n and oral routes with pgsA-sM2/*L. casei* and pgsA-CTA1-sM2/*L. casei* and challenged with the H5N2, H1N1, H9N2, H5N1 or H7N3 influenza subtypes. On 3 and 5 dpi, 3 mice were sacrificed randomly from each group, and their lung virus titers were measured using the TCID_50_ method. Mice immunized with pgsA-CTA1-sM2/*L. casei* had lower titers at 3 dpi and had significantly reduced viral replication at 5 dpi compared to mice immunized with pgsA-sM2/*L. casei* or the control groups at the same time (Fig. 5A-J). Reduced viral titers in the lungs were observed in groups of mice immunized via the i.n route relative to the mice immunized via the oral route, particularly at day 3 post infections (Fig. 5). These reduced titers may be due to routes of vaccination and challenge being the same, and the titers correlated with the survival results for lethal infections with H5N2, H1N1, H9N2, H5N1 and H7N3. Taken together, these results demonstrate that the consensus sM2 protein fused with CTA1 afforded better protection than sM2, and the i.n route was more potent than the oral route of immunization with regard to protection against a lethal challenge of divergent influenza subtypes.

Histopathology and immunohistochemistry were performed to corroborate the lung virus titer findings. At 5 dpi, lungs were randomly collected from each group of one set, fixed and stained with eosin before being examined under a light microscope. As shown in figure 5K, clear signs of profound pulmonary inflammation were observed in the lungs of mice treated with PBS or pgsA/*L. casei* for both the oral and i.n routes of administration, whereas the lungs of the mice immunized with pgsA-CTA1-sM2/*L. casei* showed no remarkable pulmonary inflammation compare to the pgsA-sM2/*L. casei*-treated mice (Fig. 5K, middle and left panel). For immunohistochemistry, immunoperoxidase method with an antibody directed against the matrix protein-2 of influenza A virus was used for the detection of virus infected cells in the respective tissues. Virus antigen in epithelial cells appears as brown coloration of the nucleus and cytoplasm. As shown in figure 5K, at 5 days p.i., numerous virus-infected cells were detected in control or pgsA-sM2/*L. casei* vaccinated mice, whereas highly reduced number of antigen positive cells were found in the mice vaccinated with pgsA-CTA1-sM2/*L. casei*, both in i.n. and orally immunized group (Fig. 5K right panel). These results indicate that mice immunized with pgsA-CTA1-sM2/*L. casei* developed immune responses that are strong enough to inhibit virus replication, which promotes the survival of mice after a lethal infection by influenza A.

### The PgsA-CTA1-sM2/*L. casei* Vaccination Induced Long-lasting Cross Protection

**Figure 6:**
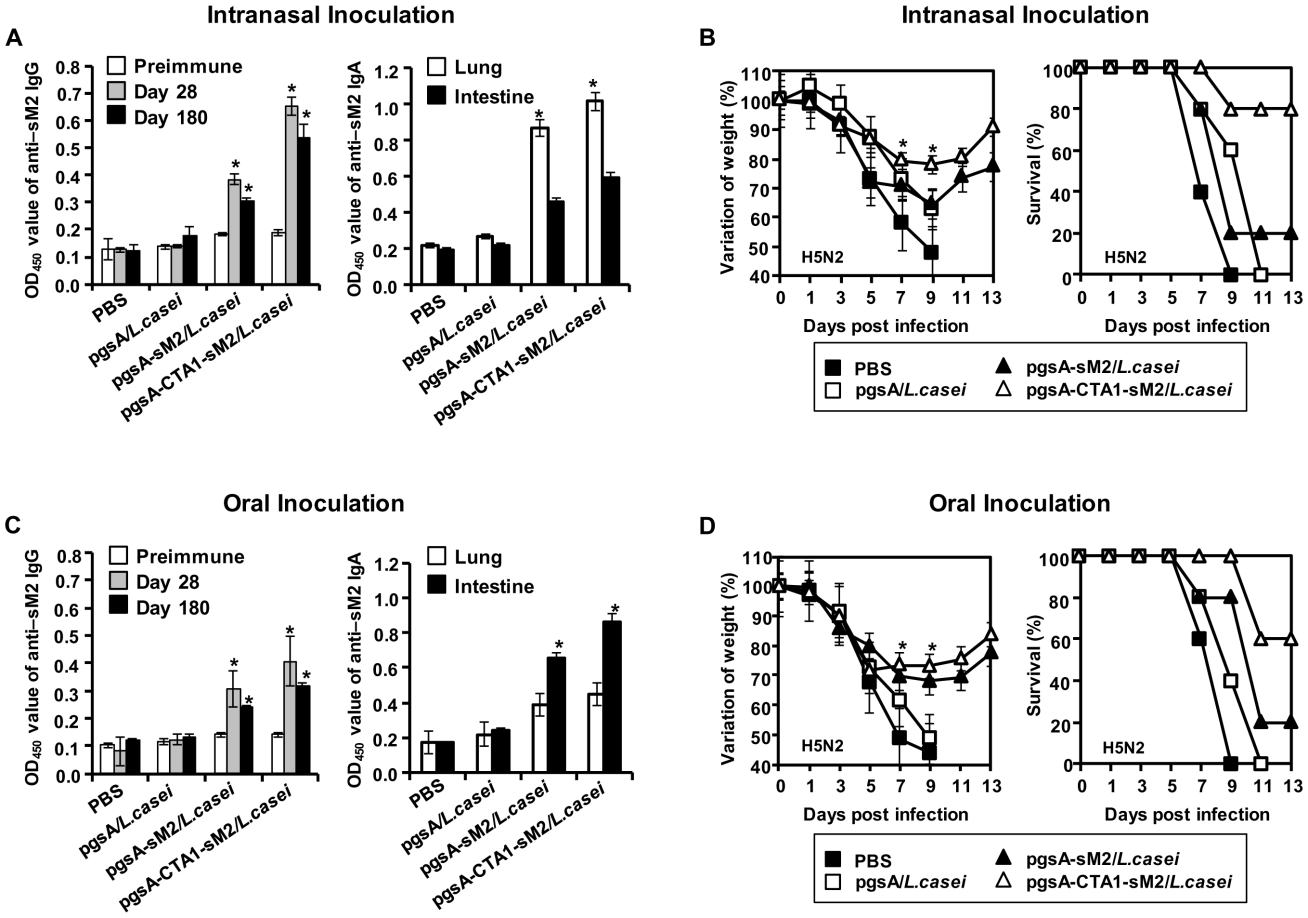
Long-lasting protection against divergent influenza subtypes. Groups of mice were immunized according to the schedule, either i.n. or orally, with PBS, pgsA/*L. casei*, pgsA-sM2/*L. casei* or pgsA-CTA1-sM2/*L. casei*. Sera were collected on days 0, 28, and 180 after the final vaccination. Lung and intestine samples were collected at day 180. ELISA was performed in triplicate using the coated sM2 protein to confirm the long-lasting antibody levels of both IgG and IgA. Mice were challenged with lethal doses of A/Aquatic bird/Korea/W81/2005(H5N2) virus (10 MLD_50_) 6 months after the final vaccination. (A and C left), The absorbance of serum IgG, (A and C right) lung and intestinal IgA specific to the sM2 protein of the intranasal (top) and oral (bottom) groups. Bars denote the means ± S.D. The asterisk indicates a significant difference between pgsA-sM2/*L. casei* or pgsA-CTA1-sM2/*L. casei* and PBS or pgsA/*L. casei* (*P<0.05). (B and D left panel) The percent of initial body weight and (B and D right panel) survival post challenge are shown. (B and D left panel) Error bars depict the standard error of the mean. *, Groups differ for weight loss, *P*<0.05 (Student's *t* test). In survival (B and D right panel), both intranasal and oral vaccination with pgsA-CTA1-sM2/*L. casei* was significantly better protected than PBS, pgsA/*L. casei*, pgsA-sM2/*L. casei* after lethal challenge with A/Aquatic bird/Korea/W81/2005(H5N2) (log-rank test, *P* = 0.01 and *P* = 0.006 respectively).

The duration of protection is an important criterion for a potential vaccine. Thus, the longevity of the immunity induced by sM2 and CTA1-conjugated sM2 were investigated by detecting serum IgG and mucosal IgA by ELISA. Significantly increase levels of sM2-specific serum IgG as well as lung and intestinal IgA were observed 180 days after vaccination (Fig. 6A and C) compare to PBS and pgsA/*L. casei* groups. Mice were challenged with A/Aquatic bird/Korea/W81/2005(H5N2), and the body weight changes and survival were monitored until 13 dpi. The unimmunized mice showed >30% body weight loss (Fig. 6B and D left panel) and died by day 9 post infection in both the oral and i.n. groups. In contrast, the mice immunized with pgsA-CTA1-sM2/*L. casei* showed negligible body weight loss, which was recovered by 13 dpi; 80% survived in the i.n. immunized group (Fig. 6B right panel), and 60% survived in the orally immunized group (Fig. 6D right panel). This result indicates that the CTA1-conjugated sM2 mucosal vaccine conferred protection against a lethal infection 6 months after the final immunization.

## Discussion

The mucosal immune system is the first immunological barrier against the pathogens that invade the body via the mucosal surface. Thus, the induction of mucosal immunity is necessary to ensure protection against multiple subtypes of influenza A virus. A respiratory virus, influenza A is responsible for annual seasonal epidemics worldwide and, occasionally, pandemics, which are caused by emerging novel subtypes/strains derived through reassortment with avian or porcine viruses. Current influenza vaccines provide strain-specific protection only. Thus, it is crucial to establish a broadly cross-protective influenza vaccine. Antigens that are well conserved among influenza A viruses are considered promising targets for the induction of cross-protection against these different subtypes. However, the goal should be the development of a first line of defense by effectively eliminating pathogens at the mucosal surface. Influenza matrix protein-2 (M2) is relatively well conserved among the influenza subtypes and can be considered a promising influenza vaccine antigen [30]. It consists of the following three structural domains: a 24-amino-acid extracellular domain, a 19-amino-acid transmembrane domain, and a 54-amino-acid cytoplasmic tail domain [39], [40]. The extracellular and cytoplasmic domains, which are well conserved among influenza viruses and play an important role in viral assembly and morphogenesis, were used in this study. Here, we developed sM2 consensus derived from the analysis of sequences of H5N1, H1N1 and H9N2 subtypes in the database. Considering the previous findings that extracellular domain particularly (aa, 1-13) is highly conserved among the influenza virus subtypes and recognized as epitope for the induction of monoclonal antibodies, which could protect influenza virus infection [56], sM2 backbone sequence from the H5N1 virus were used. For the possible homology among other subtypes we changed at the position of 14 (E–G) and 18 (R–K) and kept unchanged the conserved epitope (aa, 1–13). As shown in sequence alignment, sM2 of consensus sequence has 0–8 mismatches among the subtypes used in this study (Table 1).

Moreover, the incorporation of an adjuvant is considered essential to boost the interaction of the vaccine with the mucosal immune system [41]. Various adjuvants, such as liposomes, nanoparticles, and immunostimulating complexes (ISCOMs), have been studied and were found to improve the immune response [42], but their efficacies were not optimal. Despite its potential as a mucosal adjuvant [43], the use of cholera toxin (CT) in vaccines is limited by its innate toxicity. Thus, the toxicity of CT would have to be separated from its adjuvanticity before it could be used as a vaccine adjuvant. Studies have shown that constructs consisting of M2e fused with cholera toxin subunit A1 along with a strong ADP-ribosylating agent and a dimer of the D-fragment of *Staphylococcus aureus* protein A vaccine elicited complete protection and reduced morbidity [6], [44]. CTA1 retains the adjuvant function of CT without its toxic side effects, such as reactogenicity at the site of its administration and binding to or accumulation in the nervous tissues [45]. Based on previous findings, it has been hypothesized that the consensus sM2 fragment, when fused with the potent mucosal adjuvant CTA1, may induce broad protective immunity against divergent subtypes of influenza virus. In this study, we used the whole 22-kDa CTA1 protein (an ADP ribosyltransferase), which consists of three distinct subdomains: CTA11 (residues 1 to 132), CTA12 (residues 133 to 161), and CTA13 (residues 162 to 192). It has been reported that CTA1 lacking CTB has strong adjuvant activities without any toxicity. CTA1 enhances the IgA and IgG antibody responses, as well as CTL activity [47].

For the development of a universal mucosal influenza vaccine with a conserved sM2 peptide and potent adjuvant CTA1, recombinant *L. casei* displaying sM2 fused with or without CTA1 were constructed for mucosal delivery by the widely used live vaccine vehicle LAB [38]. The pgsA gene used in this study is an anchor for display on the surface of LAB which is derived from the pgsBCA enzyme complex of *Bacillus subtilis* and consists of transmembrane domain near its N-terminus with the domain located on the outside of the cell membrane. Thus, pgsA is able to cross the cell wall and display the heterologous protein fused to its C-terminus [17].

The developed vaccines were tested through two major routes. We found that vaccination with pgsA-CTA1-sM2/*L. casei* was able to induce a significantly higher level of sM2-specific serum IgG (Fig. 2A and B) and mucosal IgA (Fig. 2C and D) compared to pgsA-sM2/*L. casei*, and conferring protection against divergent influenza subtypes of both phylogenetic group 1 (H1, H5, H9) and group 2 (H7) [46] (Fig. 4). This study also revealed that i.n. administration was superior to the oral route of vaccination, which is consistent with other observations [48]. There may be two possible reasons to explain this phenomenon. First, the challenge route is the same as that of the vaccination; specific mucosal IgA can prevent viral colonization in the respiratory tract. Second, the volume of the inocula was 5 times lower than that for oral inoculation, which may have allowed the concentrated form of the antigen to be presented to immune cells. Because greater levels of serum IgG and mucosal IgA were detected in intranasally immunized mice than in those immunized orally (Fig. 2), an alternative explanation could be that the antigens are processed and/or presented differently to immune cells in the two mucosal compartments. Importantly, our study demonstrated for the first time that mucosal immunization with the LAB surface-displayed CTA1-conjugated sM2-based vaccine candidate induced broad protection against challenge with divergent influenza subtypes.

However, the mechanism by which Abs against sM2 mediated this broad protection is not fully understood. Previous studies have demonstrated that Abs to the N-terminus of M2e, particularly positions 1–10, inhibited the replication of the influenza A virus [49], [50]. Other studies revealed that anti-M2e IgG-mediated cellular cytotoxicity or phagocytosis can induce the removal of infected cells before progeny virus budding and spread [54], [55] which is supporting our findings of lung virus titer and immunohistochemistry data detected at 5 dpi in our challenge experiments. Therefore, in this study, combination of those responses and Abs to the N-terminus of the sM2 sequence which is conserved among the challenge viruses (Table 1) may protect the divergent influenza subtypes after mucosal immunization with the recombinant LAB CTA1-conjugated sM2-based vaccine candidate. Moreover, the cellular immune response plays an important role in controlling viral replication. We examined the Th1-type (IFN-γ) and Th2-type (IL-4) cytokine responses by the ELISPOT assay. Significantly higher levels of IFN-γ were detected in response to stimulation with both the sM2 protein and M2 peptide in mice immunized with pgsA-CTA1-sM2/*L. casei* compared to the levels in mice in the pgsA-sM2/*L. casei* and control groups (Fig. 3A and C). Similarly, substantially high levels of IL-4 were observed in mice immunized with pgsA-CTA1-sM2/*L. casei* upon stimulation with the sM2 protein and M2 peptide (Fig. 3B and D). These results further support the findings that the antibodies and cell-mediated cytotoxicity were specific to the M2 antigen and that their anti-viral activities were induced by monomeric M2, three copies of M2 fused with ASP-1 [34], [51], [52]. Together, these results indicate that sM2 adjuvanted with fused CTA1 induced immune responses in mice, which protected them from divergent influenza subtypes. In this regard, our results have significance for the use of CTA1, which has adjuvant function, in vaccine candidates.

As clinical protection is not the only parameter by which vaccine performance is assessed, we evaluated the immunogenicity of the recombinant LAB vaccine on the basis of other parameters, such as the reduction of pathological lesions and virus shedding. In this study, low titers of the challenge virus were titrated from the lungs after vaccination with pgsA-CTA1-sM2/*L. casei*, whereas challenge virus could be detected at higher titers in the mock mice and those vaccinated with pgsA-sM2/*L. casei* (Fig. 5A–J). Reduced gross and histopathological lesions consistent with viral infection are the primary parameters indicative of influenza vaccine efficacy. Here, we demonstrated that vaccination with pgsA-CTA1-sM2/*L. casei* remarkably limited the severity of the damage by inhibiting viral replication and the accumulation of inflammatory cells and virus antigen in the lung alveolar tissues, relative to the severity in the unimmunized mice and the mice vaccinated with pgsA-sM2/*L. casei* (Fig. 5K).

Our study further demonstrated, for the first time, that recombinant *L. casei* expressing CTA1-sM2 induced long-lasting immunity and conferred protection against lethal infections by influenza, even at 6 months after the final vaccination (Fig. 6), which is important for any successful vaccine. Similar results were observed in previous studies, in which M2 VLP conferred long-term immunity and cross protection and the antibodies in the sera and mucosal sites were long lived [53], [54].

In conclusion, our findings revealed that the mucosal immunization of mice with recombinant *L. casei* expressing CTA1-conjugated sM2 can induce systemic and local, as well as cell-mediated, immune responses against divergent influenza virus subtypes. Thus, the recombinant *L. casei* expressing CTA1-conjugated consensus sM2 mucosal vaccine may be a promising vaccine candidate for influenza pandemic preparedness.
